# From Practice to Reflection: A Systematic Review of Mechanisms Driving Metacognition and SRL in Music

**DOI:** 10.3390/jintelligence13120162

**Published:** 2025-12-09

**Authors:** Yinghui Wang, Mengqi Zhang, Huasen Zhang, Xin Shan, Xiaofei Du

**Affiliations:** 1 The Graduate School Arts & Culture, Sangmyung University, Seoul 03016, Republic of Korea; wyh1015@sangmyung.kr (Y.W.);; 2 The Graduate School & Education, Sangmyung University, Seoul 03016, Republic of Korea; 3 School of Mechanical Engineering, Nanjing Institute of Technology, Nanjing 211167, China

**Keywords:** metacognition, self-regulated learning, music education, music teaching, instructional interventions, strategy teaching, structured learning support, technology-enhanced intervention, meta-analysis

## Abstract

Metacognition and self-regulated learning (SRL) are widely recognized as key mechanisms for academic achievement and skill development, yet in music education they have rarely been examined through explicit instructional interventions to enable causal testing and effect evaluation. To address this gap, this study followed PRISMA guidelines and conducted a systematic review of 31 studies (including seven for meta-analysis) to identify intervention types and mechanisms, and to quantify their overall effects and moderating factors. Results indicate the following: (1) the intervention ecology is grounded in structured learning support (SLS), frequently combined with strategy teaching (ST) or technology-enhanced interventions (TEI), with full integration concentrated at the university level. (2) The mechanisms operate primarily along four pathways: structure facilitates a “plan–practice–reflection” loop, strategy instruction makes tacit experience explicit, technological feedback provides a third-person perspective, and teacher support stabilizes motivation. (3) The meta-analysis revealed a significant positive medium effect overall. (4) Intervention structure moderated outcomes, though not as a single or stable determinant. (5) Effects followed a U-shaped pattern across educational stages, strongest in secondary school, followed by university, and weaker in preschool and primary. Future research should employ proximal, task-aligned measures, conduct parallel multi-indicator assessments within the same stage, and expand evidence for multi-mechanism integration in primary and secondary school contexts. Experimental designs manipulating levels of SLS are needed to test whether ST + TEI remain effective under low-structure conditions, thereby identifying the minimum structural threshold. Extending samples to informal and professional music learners would further enhance robustness and generalizability.

## 1. Introduction

Metacognition and self-regulated learning (SRL) are widely regarded as critical abilities for successful learning (Dent and Koenka 2016). From the perspective of contemporary theories of intelligence, these regulatory abilities are also central components of intelligence rather than merely auxiliary learning skills. In Sternberg’s triarchic theory of human intelligence, so-called metacomponents—higher-order executive processes that plan, monitor, and evaluate one’s cognitive activity—are proposed as a key aspect of intelligent behavior (Sternberg 1984). In this view, intelligence is conceptualized not simply as the possession of knowledge, but as the capacity to regulate and strategically deploy one’s cognitive, motivational, and behavioral resources to achieve personally and culturally valued goals in context. Because metacognition and SRL both concern the monitoring and regulation of cognition, motivation, and behavior in goal-directed learning, they can be understood as domain-specific manifestations of such intelligent self-regulation in educational settings. Numerous studies have demonstrated that learners with stronger metacognitive and SRL abilities achieve better academic performance, transferability, and learning persistence (Heaysman and Kramarski 2022; Tang 2019). However, the development of metacognition and SRL is not entirely spontaneous; rather, it is a gradual process requiring external support (Dignath and Büttner 2008). Therefore, learners across disciplines often need instructional interventions to cultivate these abilities. To ensure clarity, it is useful to differentiate these core constructs: metacognition refers to ‘cognition about cognition,’ involving knowledge and regulation of one’s cognitive processes (Flavell 1979), whereas SRL is a broader construct that integrates metacognition with motivational, affective, and behavioral components to achieve learning goals (Schunk and Greene 2018; Zimmerman 2002). While conceptually distinct, they are deeply intertwined in practice, as effective SRL heavily relies on robust metacognitive monitoring.

Unlike other subjects, music education is highly practice-oriented and skill-dependent. Music learners must engage in substantial independent practice outside of class, and the effectiveness of practice depends not only on the time invested but also on whether learners can set goals, monitor execution, and engage in timely reflection and strategy adjustment—core manifestations of metacognitive and SRL abilities (Li et al. 2023; Yu 2023). Yet the challenge of music learning lies in the fact that, on the one hand, teachers have limited class time and cannot supervise students’ after-class practice continuously. On the other hand, students often fall into inefficient repetitive playing during independent practice, resulting in practice time not translating into effective skill improvement (Austin and Berg 2006). Thus, music education particularly requires instructional interventions to foster learners’ SRL and metacognition, enabling them to remain focused, reflective, and strategic in complex skill practice.

Nevertheless, our searches in databases such as WoS, Scopus, ERIC, and PsycINFO revealed results consistent with the observations of Li et al. (2023): although numerous studies involve SRL and metacognition, their primary goal is often to use them as strategies to enhance musical skills rather than to focus directly on the cultivation of “metacognition” or “SRL” as abilities in their own right. Hence, it is necessary to systematically review the types of interventions and their combination patterns in music education contexts.

A closer examination of the existing literature reveals a predominant emphasis on the effectiveness of interventions, with comparatively less attention paid to their underlying mechanisms. For instance, Bentley et al. (2023) reported that rhythm-based training improved self-regulation among preschoolers, yet the mechanisms driving this improvement were not explicitly unpacked. Similarly, although Miksza et al. (2018) documented enhancements in goal setting and strategy use, it remained unclear whether these gains stemmed from external guidance or intrinsic motivation. As McDonald et al. (2006) argued, without a mechanistic understanding, educational interventions are unlikely to be sustainably generalized across contexts.

On the other hand, most current studies are based on small-sample qualitative designs (López-Íñiguez and McPherson 2020; Miksza et al. 2018; Nielsen 2015; Osborne et al. 2021), which, despite reporting positive outcomes, offer limited generalizability and do not provide stable effect size estimates. This lack of comparability presents challenges for practitioners seeking evidence-based benchmarks. In this context, meta-analysis serves as a valuable approach to quantitatively synthesize the existing body of evidence and assess both the overall effectiveness and the robustness of inferences drawn from prior research (McPherson and Zimmerman, 2011).

Moreover, intervention effects may vary depending on moderating factors. Findings from general education research suggest that elementary school learners benefit more from structured and explicit teacher support, whereas secondary school students are more responsive to interventions that emphasize reflective thinking and strategy integration (Dignath and Büttner 2008). Whether similar moderating effects hold in music education, however, remains an open empirical question. Although previous reviews have addressed SRL interventions in general education or focused on particular aspects of SRL within music learning, a comprehensive synthesis that integrates intervention types, underlying mechanisms, and key moderators specifically within the context of music education is still lacking.

To address this multidimensional gap, the present study focuses on mapping the landscape of intervention types, identifying mechanistic pathways, and examining moderating variables. Methodologically, this study adopts an integrative approach combining systematic qualitative synthesis and meta-analysis: the former is used to construct an ecological framework of intervention types and a conceptual model of mechanisms, while the latter quantitatively aggregates intervention outcomes. This dual-track design seeks to balance theoretical depth with empirical breadth, ultimately offering a more comprehensive, nuanced, and practically actionable evidence base for the field of music education. Accordingly, this study addresses the following two central research questions and their sub-questions:

RQ1: What is the landscape of pedagogical interventions aimed at enhancing metacognition and SRL in music education?

(a) What types and combinations of interventions have been implemented?
(b) Through which mechanisms do these interventions enhance metacognition and/or SRL?

RQ2: What is the overall effectiveness of these interventions, and what moderating factors influence their outcomes?

(a) What is the overall effect size of interventions on metacognition and/or SRL?
(b) Does the degree of instructional structuring moderate intervention outcomes?
(c) Do educational stages significantly affect intervention effectiveness?

## 2. Theoretical Framework

Self-regulated learning (SRL) is a foundational construct in educational psychology, defined as the process through which learners actively organize their cognitive, metacognitive, motivational, and behavioral resources to achieve academic goals (Schunk and Greene 2018). According to Zimmerman’s (2002) widely recognized cyclical model, SRL comprises three interrelated phases: the forethought phase, which involves task analysis and goal setting; the performance phase, which emphasizes self-control and self-monitoring; and the self-reflection phase, where learners evaluate outcomes and attribute causes, thereby informing future learning cycles. The essence of this process lies not only in learners’ acquisition of learning strategies but more crucially in their ability to strategically activate, monitor, and adapt these strategies to achieve continual self-optimization. This model has been explicitly applied in music education, where expert performers demonstrate advanced SRL by systematically planning their practice, monitoring performance in real time (e.g., tuning, rhythm), and engaging in reflective evaluation using tools such as audio or video recordings (Dienlin et al. 2021).

A key methodological decision in the current study was to synthesize metacognition and SRL into a single effect size in the meta-analysis. This decision was grounded in a critical examination of contemporary SRL theories. As reviewed by Panadero (2017), across six influential SRL models, metacognition is consistently positioned as the central engine of self-regulation, despite variation in terminology and structural stages. Metacognition includes both metacognitive knowledge (awareness of self, tasks, and strategies) and metacognitive regulation (planning, monitoring, and evaluating), and these processes constitute the core mechanisms through which learners navigate broader SRL cycles (Flavell 1979; Schraw and Moshman 1995).

Panadero’s (2017) comparative analysis further clarifies when it is conceptually appropriate to distinguish SRL from metacognition. Such a distinction is warranted when the research aims to differentiate between learners’ declarative knowledge about cognition (i.e., metacognitive knowledge) and the broader self-regulatory process that includes motivational and behavioral components. However, when the research focus is on how learners operationally monitor and control their learning in real time—as is the case for most interventions included in this review—integrating the two constructs becomes theoretically and methodologically justified.

This conceptual overlap also extends to the measurement level. Prominent SRL measurement tools, such as the Online Self-Regulated Learning Questionnaire (OSLQ) and the Self-Regulated Learning Interview Schedule (SRLIS), although designed to assess SRL broadly, incorporate explicit evaluation of metacognitive processes (Barnard et al. 2009; Zimmerman and Pons 1986). Therefore, metacognition is not only embedded within SRL theoretically but also tightly aligned with it empirically through shared measurement structures.

In light of these theoretical justifications and empirical convergences, we integrated metacognition and SRL into a unified analytic construct in our meta-analysis. This approach ensures coherence between our conceptual framework and analytic strategy, enhancing the robustness and interpretability of our findings.

## 3. Methods

### 3.1. Reporting Guidelines and Protocol Registration

This study was conducted and reported in strict accordance with the Preferred Reporting Items for Systematic Reviews and Meta-Analyses (Page et al. 2021). To ensure traceability, reproducibility, and transparency, all stages of searching, screening, data extraction, and synthesis were cross-checked against the PRISMA checklist. Any deviations and their justifications were documented during the implementation process. In addition, a scoping search of PROSPERO and major academic databases using relevant keyword combinations revealed no ongoing or published reviews addressing the same research question, namely instructional interventions in music learning contexts with SRL or metacognition as outcome measures.

### 3.2. Information Sources and Search Strategy

In early August 2025, we systematically searched four core databases: Web of Science (Core Collection), APA PsycINFO, ERIC, and Scopus. The search strategy was finalized after multiple rounds of trial searches and thesaurus expansion, combining free-text terms and subject headings, with Boolean logic applied to balance breadth and precision. The final cross-database search string was as follows: music AND (education OR instruction OR teaching OR learning OR classroom OR training OR strategy OR pedagogy OR curriculum) AND (“learning regulation” OR “self-regulated learning” OR “self-regulation” OR “SRL” OR “goal setting” OR “cognitive regulation” OR “time management” OR “learning strateg*” OR “motivational regulation” OR metacogniti* OR “metacognitive strategy” OR “self-monitoring”). Ultimately, the initial database check yielded a total of 1825 records (for full search details, see Supplementary Materials Table S1).

### 3.3. Eligibility Criteria

To ensure methodological quality and alignment with the objectives of this review, all 1825 retrieved records underwent a two-stage screening process, based on the following inclusion criteria:

(1) Publication type: Only peer-reviewed journal articles were included. Book chapters, conference abstracts, dissertations, unpublished manuscripts, and retracted publications were excluded.
(2) Publication timeframe: To ensure the currency and relevance of findings, eligible publications were restricted to those published between 2015 and 2025. This ten-year span reflects the latest developments in teaching interventions and the measurement of self-regulated learning (SRL) and metacognition in music education. It encompasses the period during which SRL and metacognitive models have matured, the emergence of technology-enhanced interventions (e.g., video-based self-feedback and digital monitoring platforms), and the evolution of strategy support modes in post-pandemic remote/hybrid learning contexts. As such, the selected timeframe holds strong contextual and review value.
(3) Language: Only articles published in English were considered, to ensure consistency across databases and international accessibility.

Based on the above criteria, 810 records were excluded during the initial screening phase. The remaining 1015 records were retained for further eligibility assessment. The final database search and data export were completed on 7 August 2025. On that date, all remaining records were exported and compiled into a unified Excel sheet for subsequent manual screening. Subsequently, the two primary researchers, Y.W and M.Z independently conducted the screening. In cases of disagreement, the third author, X.S, intervened as an arbitrator. The entire process was carried out in two stages. To ensure the reliability of the screening and coding process, we calculated and reported the inter-rater agreement, which yielded κ = 0.78 (95% CI [0.70, 0.86]), indicating a substantial level of agreement among the reviewers.

Stage one (title and abstract screening): The key criterion was whether the study involved instructional interventions or strategies in music contexts, with SRL or metacognition explicitly measured as a primary or secondary outcome variable. Studies failing to meet this threshold were excluded (see Table 1). For instance, although some studies incorporated SRL/metacognition strategies, they treated them as independent variables rather than outcomes—such as Lorenzo de Reizábal and Benito Gómez (2019), which applied SRL strategies to improve students’ professional skills but did not directly assess improvements in SRL or metacognition. After this stage, 65 studies were retained for full-text review.

**Figure 1:**
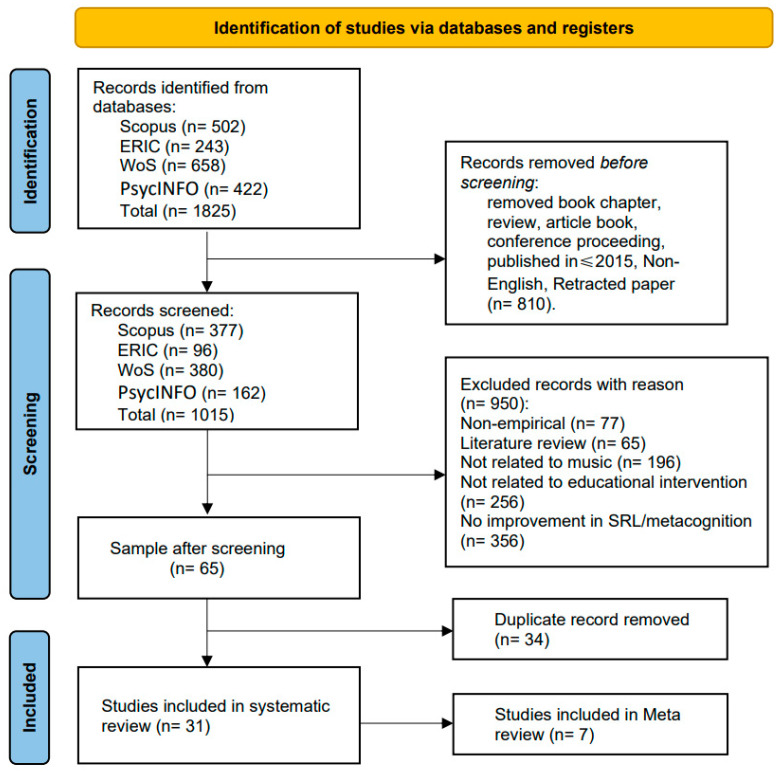
PRISMA diagram.

Stage two (full-text screening and deduplication): The 65 full-text articles were reviewed for inclusion, with a focus on the alignment between their research design and conclusions and the objectives of this study. Simultaneously, duplicate records were identified and removed. While all 65 articles met the inclusion criteria, 34 were identified as duplicates. After deduplication, 31 unique studies were ultimately included in the systematic review (see Figure 1). A complete list of the 65 full-text articles reviewed is provided in Supplementary Materials Table S2.

In total, only studies among the 31 included that reported calculable effect sizes, or from which effect sizes could be extracted and converted, were further included in the meta-analysis (*n* = 7).

### 3.4. Data Extraction and Management

Two reviewers independently extracted data from the eligible studies using a predefined extraction form, with verification by the first author. Disagreements were resolved through discussion or arbitration by a third reviewer. Extracted fields included the following: (a) general information—title, authors and year, country/region, educational stage, sample size and gender composition, study design; (b) intervention details—content components, implementer qualifications, frequency and duration, follow-up; (c) intervention type and structuring level; (d) measurement methods; (e) effect sizes; (f) funding sources and conflicts of interest (COI); and (g) risk of bias (RoB). All data were entered and stored in Excel for centralized management and subsequent analysis.

### 3.5. Coding of Intervention Types, Structural Intensity, and Educational Stage

#### 3.5.1. Intervention Type Coding

To compare the mechanisms of different instructional approaches, each intervention was categorized into one of three types: (1) Strategy Teaching (ST)—explicit instruction of strategies and metacognitive procedures; (2) Technology-Enhanced Interventions (TEI)—technology used as the primary medium or trigger; (3) Structured Learning Support (SLS)—external scaffolds such as scripts, checklists, schedules, standardized materials, or monitoring protocols, which constrain task organization and execution without direct strategy instruction or exclusive reliance on technological feedback.

**Figure 2:**
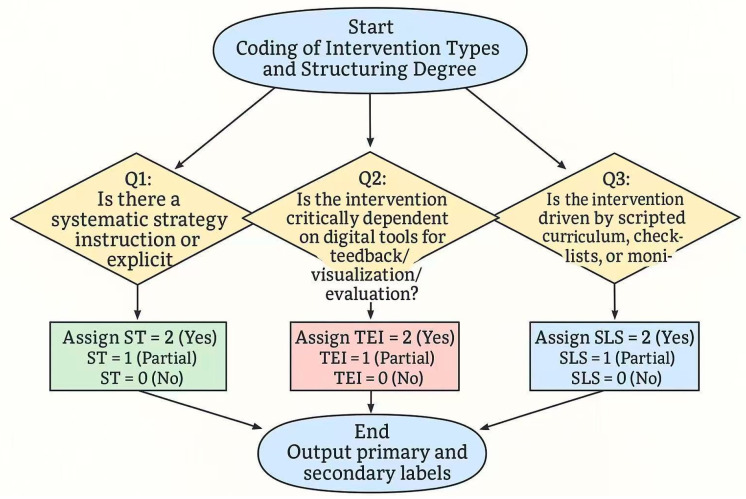
Intervention Type Coding Process.

For studies employing multiple approaches, the “core component” most central to the intervention’s mechanism was coded as the primary label, while others were recorded as secondary features to ensure comparability and consistency. Structuring level was coded independently from intervention type (see Figure 2).

Two independent coders followed these rules; disagreements were discussed by comparing the weight of intervention elements, and unresolved cases were adjudicated by a third reviewer. The highest-scoring type was retained as the primary label, with other significant components recorded as secondary. For intervention type coding, inter-coder agreement reached κ = 0.76, 95% CI [0.68, 0.84], indicating a high level of reliability.

#### 3.5.2. Structuring Level Coding

In addition to intervention type, structuring level was coded to reflect the strictness of external organization and implementation constraints. High structuring referred to interventions with standardized teacher training or scripts, fixed frequency and duration, standardized teaching materials, and adherence monitoring. Moderate structuring included relatively fixed procedures or checkpoints, but allowed flexibility in implementation and practice content. Low structuring referred to general guidance or simple frameworks, lacking enforced execution or systematic monitoring. For structural intensity coding, inter-coder agreement was *κ* = 0.82, 95% CI [0.75, 0.89], further demonstrating the robustness and stability of the coding process.

#### 3.5.3. Coding of Educational Stage

In this review, educational stage was coded according to the descriptions provided in the original studies. Specifically, CÉGEP level, undergraduate, and postgraduate programs were uniformly categorized under “university level” to reflect the broader context of higher education. Preschool, primary, secondary, and high school were coded separately. Studies spanning multiple stages were coded as indicated by the authors.

### 3.6. Data Synthesis

#### 3.6.1. Qualitative Synthesis

This study employed a mixed-method synthesis approach to integrate findings from both qualitative and quantitative studies. A convergent synthesis design was adopted, in which two independent streams of analysis were conducted in parallel to address distinct research questions. First, all 31 studies meeting the inclusion criteria were subjected to a qualitative thematic synthesis to address RQ1. Second, a subset of studies (*n* = 7) that provided sufficient data for effect size calculation was included in a quantitative meta-analysis to address RQ2.

#### 3.6.2. Meta-Analysis

Among the 31 studies included in this review, only seven provided computable effect sizes; the remaining studies were not entered into the quantitative synthesis due to missing data. To ensure robustness and representativeness, we applied the meta-analytic inverse-variance method to estimate the overall effect (Hedges and Olkin 2014), informed by practice in correlation-based meta-analyses of educational interventions (Dignath and Büttner 2008). On this basis, heterogeneity was assessed using *Q*, *I*^2^, and *τ*^2^, and subgroup analyses were conducted for RQ2. The meta-analytic procedures in this section were performed independently by the author Du.

When a single study reported multiple related effect sizes (e.g., distinct SRL indicators or subscales within the same experiment), within-study outcomes were first aggregated using the inverse-variance method to maintain statistical independence and avoid overweighting multi-outcome studies. To verify that this pooling approach did not bias the summary estimate, we additionally employed cluster-robust variance estimation (RVE) (Hedges et al. 2010). The RVE model yielded a highly comparable pooled effect (*g* = 0.57, 95% CI [0.28, 0.85]) to the primary random-effects analysis, confirming that the observed mean effect was robust to potential dependence among within-study outcomes.

We also conducted a leave-one-out (LOO) influence analysis as a form of sensitivity analysis to examine the susceptibility and stability of the overall effect estimate to single-study influence. Each point represents the pooled effect size (*g*) after omitting one study, with the dashed vertical line indicating the overall pooled estimate from the full sample (*g* = 0.56). The analysis shows that removing any single study did not substantially alter the summary effect (|Δ*g*| ≤ 0.10), and all confidence intervals remained above zero. The most influential case was study 11 (S11); excluding it reduced the pooled estimate to *g* = 0.46, yet the overall interpretation of a positive medium effect remained unchanged. This confirms the robustness of the meta-analytic result against single-study influence.

### 3.7. Risk of Bias Assessment

The Joanna Briggs Institute (JBI) critical appraisal tools were used to assess methodological quality and risk of bias for the included studies (Aromataris et al. 2024). Cross-sectional, quasi-experimental, and qualitative studies were evaluated with the corresponding checklists. Each study was assessed independently by two reviewers, and final judgments (low/moderate/high risk) were reached through discussion. Given the limited number of relevant studies and the dual objectives of conducting both a systematic review and a meta-analysis, all eligible studies were included in the systematic review. Only those rated as low or moderate risk were considered for meta-analysis. Individual RoB item scores and justifications for each decision were fully documented.

### 3.8. Funding and Conflicts of Interest

Funding sources and COI statements were systematically examined, and the potential influence of sponsors on research questions, design, analysis, or reporting was assessed. No evidence was found of direct sponsor involvement in methods or interpretation of results; therefore, no studies were excluded on the basis of funding.

## 4. Results

### 4.1. Descriptive Findings

The characteristics of the included studies were summarized and reported according to the following categories: Study ID, Author (Year), Country, Method, Participants (*n*), Educational stage, SRL/Metacognition-related results, Intervention type, Structuring level, and Risk of bias. Table 2 presents all included studies, their key information, and main findings, allowing cross-study comparison and evidence synthesis.

A total of 31 studies were included in this review. In terms of research design, multi-case qualitative studies (*n* = 7) and quasi-experiments (*n* = 6) were the most common. This distribution reflects both the practical constraints of music education contexts and the characteristics of the participants: since SRL and metacognition are highly process-oriented and context-dependent, researchers often preferred multi-case and micro-level qualitative approaches to capture learning trajectories in detail. Moreover, in authentic classroom settings, randomized controlled trials were difficult to implement due to limitations of scheduling, school–family coordination, and classroom management; as a result, teacher-led quasi-experiments provided a more feasible research design.

With regard to sample size, the 31 studies involved a total of 2593 participants. The overall distribution exhibited a pattern of “many small-sample studies but large-sample studies contributing disproportionately”. Specifically, 13 studies had small samples (≤20 participants), 13 studies had medium samples (41–140 participants), and only 5 studies included large samples (≥171 participants), yet these accounted for nearly half of all participants (46.0%). The overall median sample size was 83, indicating that this field remains dominated by small- to medium-scale intervention and observational studies.

Geographically, the studies spanned 13 countries and regions, including three multinational investigations. The United States contributed the largest number of studies (*n* = 7), followed by Australia (*n* = 4) and China (*n* = 3). Canada, Norway, The Netherlands, and Spain each contributed two studies. Overall, the distribution of studies was concentrated in developed regions with richer educational resources, particularly in Europe, North America, and other English-speaking countries.

### 4.2. RQ1

#### 4.2.1. (a) Landscape of Intervention Types and Combinations

Among the 31 studies identified, four—although highly relevant to music learning and the development of self-regulation/metacognition and informative for understanding mechanisms—were excluded from the RQ1 analysis because they lacked identifiable instructional components that could be classified as SLS, TEI, or ST.

In the remaining 27 studies, intervention types formed an ecology anchored in SLS. SLS appeared most frequently (24/27) and was seldom implemented in isolation, typically co-occurring with ST or TEI (see Table 3 and Figure 2). The SLS × ST configuration was most common (10/27), representing the principal pathway for strengthening learners’ self-regulation; SLS × TEI ranked second (8/27), leveraging technological feedback and evidentiary logging to reinforce practice monitoring and self-calibration, whereas the ST + TEI combination (without SLS) was entirely absent in this sample. By educational stage, preschool cases were dominated by SLS (2/2), and primary schooling showed the first appearances of TEI and both dyads (SLS + ST, SLS + TEI). Moreover, although the triadic integration SLS + ST + TEI was relatively infrequent, it clustered in upper-undergraduate or professional training contexts (Li et al. 2023; Utermohl de Queiroz et al. 2025), where interventions tended to span the full practice cycle and were implemented with high explicitness and fine-grained orchestration, indicating greater systematicity and depth.

#### 4.2.2. (b) Mechanisms Underlying Metacognitive and SRL Enhancement

First, the central function of SLS lies in making practice goals clear, precise, and contextually adapted to students’ individual needs, enabling them to “first achieve stability” and then gradually shift attention toward “doing better”. In preschool classrooms, teachers embed rhythm and movement into daily routines so that regulatory behaviors such as waiting, turn-taking, and cooperation are naturally integrated into play (Bentley et al. 2023; Williams et al. 2023; Zachariou et al. 2023). In secondary school classrooms, structured procedural routines facilitate the habit of “setting goals first, then practicing, and finally reflecting” (Prichard 2017, 2021).

Second, the core of ST is the explicit articulation of effective learning methods that would otherwise remain implicit. Through teachers’ questioning and modeling, students gradually learn to reflect on how to proceed during practice and how to evaluate and adjust afterward. Microanalysis (Miksza et al. 2018) and OMMP (Osborne et al. 2021) are effective precisely because they integrate “goal setting, attentional control, self-evaluation, and causal attribution” into a closed loop, ensuring that each practice session becomes a traceable learning process. Similarly, Hatfield’s (2016) psychological skills training further consolidated goal setting into a transferable learning habit.

Third, TEI transforms what students believe they are doing into observable evidence of what they actually did. Audio recording (Silveira and Gavin 2016), video playback (Bae et al. 2025; Boucher et al. 2020; Miksza et al. 2018), and delayed review (Song et al. 2024) allow students to step back from a “first-person” to a “third-person” perspective, making errors easier to detect and thereby triggering more precise self-monitoring and correction. Robot-guided self-assessment further institutionalizes the loop of “scoring–explaining–rehearsing–re-evaluating,” creating a replicable practice cycle. Real-time intonation feedback software and electronic portfolio platforms extend this process by linking immediate performance to long-term records, turning practice trajectories from “visible external evidence” into “invisible internal questioning” (Prichard 2017; López-Íñiguez and McPherson 2020). However, the effectiveness of these tools depends on the degree to which teachers embed them into instructional routines and align them with repertoire demands. When integration is loose or poorly matched, effects tend to plateau rather than show sustained growth (Brook and Upitis 2015; Wan et al. 2023).

Finally, teachers’ autonomy support acts as a stabilizer within interventions. Classroom-based autonomy support is positively associated with students’ engagement in metacognitive behaviors such as planning, monitoring, and evaluating (Zachariou and Bonneville-Roussy 2024). In parallel, emotion regulation strategies such as reappraisal help students reconstruct situational meaning, reducing the cognitive load of negative emotions on learning resources (Peistaraite and Clark 2020), while mindfulness training significantly strengthens self-efficacy and self-awareness (Koner et al. 2024).

Overall, single mechanisms often yield localized or proximal improvements—such as practice checklists enhancing low-level cognitive strategies for beginners (Bae et al. 2025), or short-term strategy instruction improving practice efficiency without altering overall SRL levels (Prichard 2017). Yet they rarely transform learners’ broader SRL and metacognitive patterns. Only when multiple mechanisms are combined as an integrated set are sustained and stable gains across the entire learning cycle more likely to emerge.

### 4.3. RQ2

#### 4.3.1. (a) Overall Efficacy of Interventions

**Figure 3:**
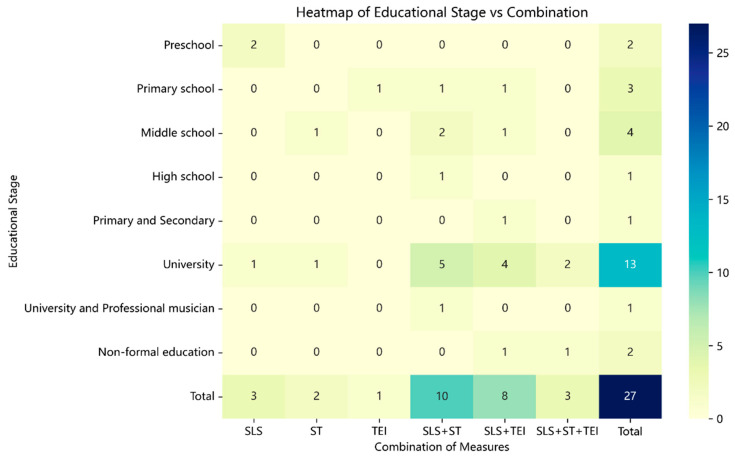
Heatmap of Intervention Combination Distribution by Educational Stage.

Overall, instructional interventions in music-learning contexts yield a moderate, positive effect on learners’ metacognition and self-regulated learning (pooled *g* = 0.563). At the level of confidence intervals (CIs), small-sample studies (e.g., *n* ≈ 16) show wider CIs and less stable estimates due to larger standard errors, whereas studies with samples in the hundreds exhibit narrower CIs and more precise estimates (see Figure 3 and Table 4). In terms of the effect distribution, most studies fall in the small-to-moderate range: small effects (*g* ≈ 0.03–0.10) are typically non-significant (*p* > 0.2–0.8;) (Dent and Koenka 2016), moderate effects (*g* ≈ 0.40–0.80) are generally significant (*p* < 0.05) (Bae et al. 2025; Boucher et al. 2020; Li et al. 2023; Silveira and Gavin 2016), and a minority report large effects (*g* ≈ 1.70–2.25; *p* < 0.001) that pull up the overall mean (Prichard 2021). This pattern aligns with pronounced heterogeneity, indicating that real differences attributable to educational stage, intervention composition, and measurement orientation exceed what would be expected from sampling error alone.

#### 4.3.2. (b) Moderator Analysis: The Role of Instructional Structuring

Across the eligible studies, the degree of structuring demonstrated a significant moderating effect on intervention outcomes, but the pattern was not strictly monotonic or linear.

**Figure 4:**
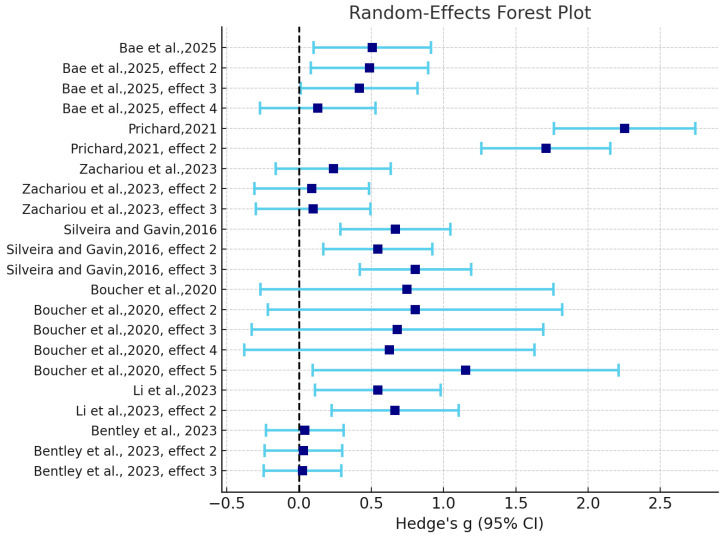
Forest Plot of Effect Sizes in Included Meta Studies.

**Figure 5:**
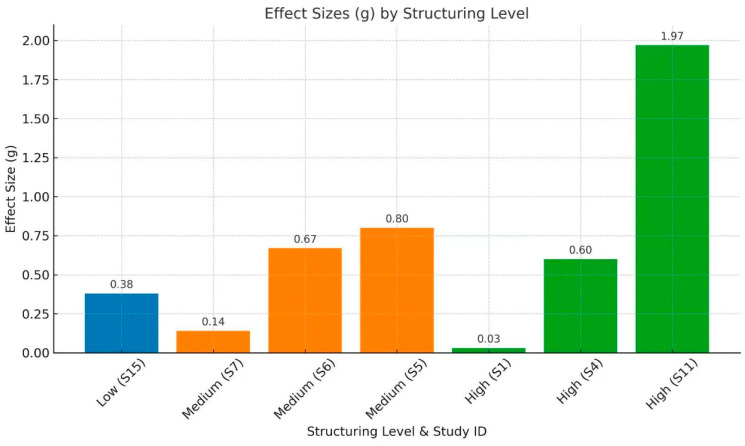
Effect Sizes (*g*) by Structuring Level in Meta-Analysis Studies.

Under low-structured conditions, where practice checklists served as the primary scaffold, gains were modest. For example, S15 (*g* = 0.383) primarily facilitated lower-order strategies such as rehearsal and organization, producing limited improvements in metacognitive self-regulation among university learners (see Figure 4 and Figure 5). In contrast, moderate structuring exhibited pronounced divergence: when interventions consisted of only simple procedures without clear goals or high-quality feedback, the effects were small, as in S7 (*g* = 0.140). This study targeted early primary school students, whose developmental stage and the limited sensitivity of measurement tools may have diluted the observed effects. By comparison, at the secondary level, once actionable feedback loops were integrated, effects rose substantially into the medium-to-large range, even when some degree of learner autonomy was retained—for example, S6 (*g* = 0.670) and S5 (*g* = 0.795).

High structuring also revealed a dual character. When structural intensity was tightly coupled with explicit strategy instruction and proximal measures, effects were magnified substantially, as in S11 (*g* = 1.971) and S4 (*g* = 0.604). Yet in preschool contexts, if structuring devolved into mechanical checklists or if measurement design was misaligned, gains could be markedly attenuated, with S1 (*g* = 0.032) representing a typical case. From the perspective of confidence intervals, highly effective structured interventions generally yielded narrower intervals that did not cross zero (e.g., S11 and S4), whereas low-structured or feedback-deficient moderate interventions were more likely to produce wide or zero-crossing intervals (e.g., S7 and S15). These findings indicate that intervention effectiveness is highly sensitive to the quality of feedback, clarity of goals, and congruence between measurement and intervention.

In general, structuring appears to act as a moderating factor in interventions, but it is neither singular nor stable in its effects. Its impact depends more fundamentally on the interplay of instructional content, teachers’ implementation, learners’ developmental stage, and the quality of feedback mechanisms.

#### 4.3.3. (c) Moderator Analysis: The Impact of Educational Stages

Synthesizing evidence across educational stages, instructional interventions exert a moderate and significant positive effect on SRL and metacognitive abilities in music education (overall *g* = 0.356, 95% CI [0.266, 0.466]). The confidence interval lies entirely above zero and is relatively narrow, suggesting stable estimates and supporting the conclusion that “interventions are overall effective.”

**Figure 6:**
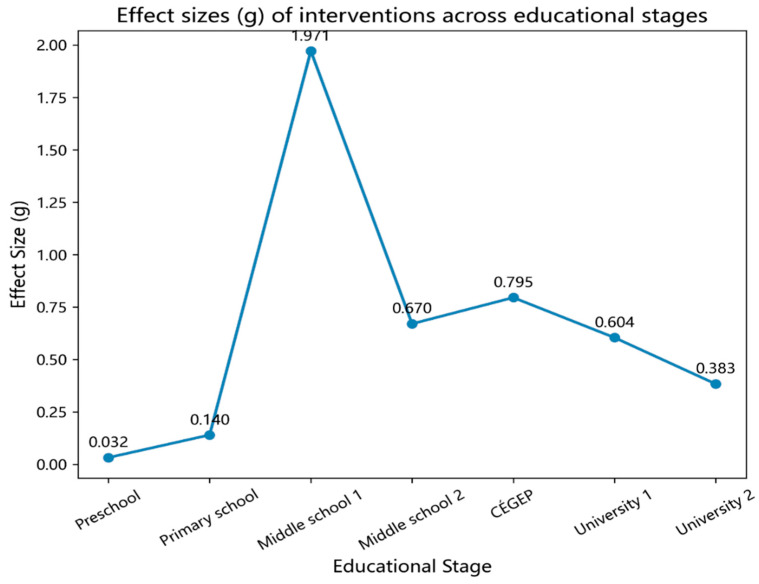
Effect Sizes Across Educational Stages.

When examined by educational stage, the effects follow an inverted U-shaped pattern (see Figure 6). The peak appears at the secondary-school level: Bentley et al. (2023) reported the largest effect (*g* = 1.971, 95% CI [1.436, 2.506]), while Silveira and Gavin (2016) also found a moderate effect in upper secondary students (*g* = 0.670, 95% CI [0.450, 0.890]). Both CIs exclude zero, indicating robust estimates. At the university level, effects were moderate but significant. Li et al. (2023) observed a relatively large effect (*g* = 0.604, 95% CI [0.294, 0.914]), whereas Bae et al. (2025) reported a smaller effect (*g* = 0.383, 95% CI [0.180, 0.585]). The divergence between the two is likely attributable to differences in intervention composition, structural intensity, feedback loops, and measurement alignment. Specifically, Li implemented an integrated, highly structured program with proximal measures of metacognitive regulation, while Bae relied primarily on a low-structured SLS checklist that did not foreground the closed cycle of goal setting, self-evaluation, and strategy adjustment, and instead assessed outcomes using the more distal MSLQ scale. As a result, improvements were mainly confined to lower-order strategies (e.g., rehearsal and organization) with limited gains in metacognitive self-regulation, leading to an overall smaller effect size. Participant characteristics and task contexts further differentiated outcomes: Li’s sample comprised preservice teachers engaged in audio playback and self-evaluation feedback, whereas Bae’s study involved novice non-music majors practicing with basic instrument checklists. These contrasts may have collectively amplified the discrepancy in effect sizes and produced divergent CI widths.

By contrast, preschool (*g* = 0.032, 95% CI [−0.124, 0.187]) and early elementary school (*g* = 0.140, 95% CI [−0.089, 0.370]) studies yielded CIs that crossed zero, suggesting that at early developmental stages, improvements are limited or measurement sensitivity is insufficient.

## 5. Discussion and Conclusions

This review synthesizes intervention and empirical research on SRL and metacognition in music-learning contexts over the past decade and highlights three broad points of consensus: First, interventions typically use SLS as a foundation complemented by ST and TEI. Second, effects follow an “inverted-U” trend across educational stages—largest in lower secondary, followed by university, and smallest in primary and preschool. Third, outcomes hinge not only on the degree of structuring, but also—often more strongly—on other factors.

Primarily, in terms of the research landscape, the 31 included studies are dominated by multi-case and quasi-experimental designs, with overall medium sample sizes and a mix of a few large and several small samples. Notably, the evidence base skews toward Europe/North America and English-speaking contexts, with scarce contributions from developing countries and the Global South. This regional concentration and imbalance in knowledge production have long been noted in international discussions of music education: global knowledge formation has been dominated by Anglo-American research traditions, underscoring the need for culturally sensitive international pathways and cross-linguistic evidence (Kertz-Welzel 2021).

Secondly, in terms of intervention composition and underlying mechanisms, the use of SLS was nearly ubiquitous, though most often implemented in conjunction with ST or TEI. The SLS × ST configuration emerged as the most common, followed by SLS × TEI. While the triadic integration of all three approaches was relatively rare, the dyadic combination of ST and TEI was entirely absent from the sample. Mechanistically, sequenced tasks and clear goal setting provide a stable practice frame that helps students convert piecemeal practice into sustainable habits, aligning with Prichard’s (2021) findings on secondary-level music practice. Making tacit practice know-how explicit through teacher guidance and modeling supports students’ on-task monitoring and strategic adjustment (Miksza et al. 2018), echoing Hatfield’s (2016) “skill transfer” effect. In parallel, TEI augments practice by enabling a “third-person” view—via audio/video capture or real-time feedback—so that deviations are more readily detected and corrected (Boucher et al. 2020; López-Calatayud and Tejada 2024; Silveira and Gavin 2016). However, without sustained teacher embedding and interpretive scaffolding, technology effects tend to fade, consistent with Wan et al. (2023). Additionally, emotion regulation and teacher support emerge as a repeatedly verified “implicit throughline”: affective and motivational states mediate SRL gains, and reappraisal can free cognitive resources and deepen reflection (Peistaraite and Clark 2020). Overall, the findings of this study are consistent with the review by Dignath and Büttner (2008), which emphasized that a single, uniform procedural design is insufficient to sustain the long-term effects of interventions. At the same time, the role of teacher feedback should not be underestimated. As highlighted by Hattie and Timperley (2007), the quality of feedback and the manner in which it is embedded directly determine the magnitude of learning gains; effective feedback must be aligned with learners’ goals, task demands, and current states, and should function to regulate and optimize the learning process. The meta-analysis by Wisniewski et al. (2020) further substantiated and extended this perspective. Reanalyzing 435 studies, they found that the average effect of educational feedback was of moderate magnitude (*d* = 0.48) and accompanied by substantial heterogeneity. Their findings indicate that the effectiveness of feedback is not constant but depends on its information richness: feedback that simultaneously provides guidance at the task, process, and self-regulation levels is the most effective.

Meanwhile, our analysis also revealed several cases of apparent inconsistencies across studies. Specifically, in early educational stages (e.g., preschool and early elementary), interventions were often highly structured, yet yielded relatively small effect sizes. Such discrepancies may not necessarily reflect ineffective interventions but could rather indicate a measurement–intervention mismatch—a misalignment between the intervention targets and the tools used to assess them. For instance, in Bentley et al. (2023), a context-rich rhythmic intervention was designed to promote task-based self-regulation among preschool children (e.g., real-time adjustment and sustained attention). However, the outcomes were primarily assessed using domain-general executive function tasks, global teacher evaluations, and verbal interview data. For young children whose self-regulation behaviors are often expressed nonverbally and contextually, assessment methods requiring verbal articulation of “how I did it” may exceed their developmental capacities, potentially leading to an underestimation of intervention effects. Similarly, Zachariou et al. (2023) highlighted the unequal visibility of intervention components across different evaluation tools: teacher ratings tend to be more sensitive to motivational and emotional changes, while classroom observations better capture behavioral and strategic regulation, which may result in divergent conclusions. Consequently, if the selected assessment tools do not align with the proximal mechanisms activated by an intervention, its effects may be systematically underestimated, particularly in younger populations (Zachariou et al. 2023). To support both instructional design and methodological rigor, we further present in Supplementary Materials Table S3 a mapping of the measurement instruments used in each study, along with their alignment to the intended SRL/metacognitive mechanisms (proximal vs. distal). This mapping enables a more transparent evaluation of the intervention–measurement fit, and helps identify potential sources of bias in effect size estimation across studies.

From the overall effects, we observed an inverted-U pattern peaking at lower secondary school. These observations align with the view of a “stage-sensitive window,” whereby adolescence marks a new sensitive phase for SRL development: expanding cognitive structures and social experiences gradually support more mature regulation (McClelland et al. 2010). However, between-study heterogeneity is substantial. The reported 95% CI indicates considerable variability of true effects across contexts and samples; LOO sensitivity analyses show that certain large-effect studies (e.g., S11) influence the pooled estimate, yet their exclusion does not alter the interpretation of a medium positive effect. Accordingly, we remain cautious in extrapolating the inverted-U pattern and the moderating role of structure, particularly given the paucity of high-school evidence; larger, methodologically rigorous studies are needed to reduce uncertainty and test robustness across contexts. In addition, with respect to “degree of structuring,” our evidence supports a “necessary-but-not-sufficient” stance: when routines, modeling, and fixed content are coupled with explicit strategy instruction, structured reflection opportunities, and high-quality feedback, structuring becomes the track that activates SRL/metacognitive cycles—as seen in the pronounced impact of secondary-level practice-instruction interventions (Prichard 2021). Conversely, when structuring devolves into mechanical checklists without explicit strategy and reflection—or when evaluation relies on insensitive, distal indicators—effects can be dampened or effectively “invisible,” even with high implementation fidelity (Boucher et al. 2020). More broadly, structuring likely functions as a moderator, but not a single, stable one; its leverage depends on content, teacher enactment, learner age/developmental stage, and feedback quality.

We also note several additional, novel observations. Miksza et al. report that students with lower SRL baselines show the largest gains in goal setting, strategy use, and self-monitoring, whereas higher-baseline students primarily clarify and consolidate existing strategies. Building on this, future work could compare two “low-baseline” groups—primary-level beginners and university-level novices—under comparable task difficulty and feedback frequency to test who improves faster and transfers more robustly, and whether tiered instruction and differentiated feedback amplify this “low-start, high-gain” effect.

Finally, while this review elucidates the integrated value of teaching interventions for advancing SRL and metacognition in music learning, current evidence is subject to several constraints. First, this study included only peer-reviewed literature published in English, which may introduce a degree of language bias. On one hand, this criterion may have excluded valuable studies published in other languages, particularly those from non-English-speaking countries with strong traditions in music education (e.g., Germany, France), as well as emerging research communities in Asia (e.g., China, South Korea). On the other hand, due to the high concentration of academic publishing in English, the included literature tends to be dominated by studies conducted in Western, English-speaking countries (e.g., the United States, United Kingdom, Canada, and Australia). This sampling bias may limit the cultural and educational diversity of the included interventions and potentially reduce the cross-cultural applicability and external validity of the findings. Second, although the literature search window was defined as 2015–2025, the final database search and data export were completed on 7 August 2025. This temporal gap implies that, owing to the inherent lag between publication and database indexing, relevant studies published in the final months of 2025 or released online around that cutoff date may not have been fully captured in the present search. Third, the limited number of studies eligible for the meta-analysis attenuated the statistical power of subgroup analyses—particularly given the absence of high-school evidence—rendering stage-specific pooled effects potentially sensitive to single-study influence. The small *k* also constrained the assessment of publication bias. Consistent with methodological consensus, when *k* < 10, tests such as Egger’s regression and trim-and-fill are underpowered and therefore uninformative, with results easily driven by individual studies and potentially misleading (Sterne et al. 2008). Consequently, we cannot accurately quantify the potential impact of publication bias using statistical tests alone. To address these uncertainties cautiously, we additionally report 95%CI to highlight the high heterogeneity and conduct LOO analyses, which corroborate the robustness of the core findings. Nevertheless, while these procedures gauge single-study influence, they cannot eliminate the fundamental limitations imposed by the small evidence base. Finally, within music education, rigorous RCTs and meta-analytic samples suitable for pooling remain limited; accordingly, we adopted a narrative synthesis to surface “mechanism–context–stage” regularities and offer an analytic scaffold for future research.

## 6. Implications for Music Education Practice

The findings provide an evidence-informed, stage-specific roadmap for classroom and studio practice in music education. Interventions targeting metacognition and SRL are not “one size fits all”; rather, their design should be closely aligned with learners’ developmental stage.

Drawing on the 31 studies included in this review, we distilled minimum structuring thresholds (MSTs)—the least amount of scaffolding needed to sustain the effectiveness of ST and TEI when SLS is reduced. The MST represents an empirically inferred “minimal viable set” of structures that keeps the SRL cycle of planning, monitoring, and reflection intact.

At the preschool and primary school stages, where linguistic abilities, abstract reasoning, and self-awareness are still developing, both ST and TEI require highly structured conditions to be effective. For ST, this entails embedding simple, guided reflective prompts within teacher-led musical play that is strongly scaffolded (e.g., S7). For TEI, it involves tools that provide immediate sensory feedback (e.g., S10; S8) to create developmentally appropriate, rapid micro-cycles of enactment–monitoring–adjustment. At this stage, structure must be dense and externally driven.

At the secondary school stage, as abstract thinking matures, the MST evolves. For ST, effective interventions typically include mandatory periodic written cycles of planning and reflection (e.g., structured practice logs) that require learners to make strategy selection and performance outcomes explicit (e.g., S11, S23, S24). For TEI, the threshold is characterized by evidencing the practice process and binding it to reflective work—most commonly by requiring structured self-evaluation and feedback integration based on audio/video records (e.g., S6). Compared with the immediacy emphasized in younger cohorts, the MST at this stage privileges the representation, scrutiny, and analysis of one’s own performance, marking a shift from the learner as “observer” to “interpreter”.

At the university level, autonomy becomes paramount, and the MST is leaner yet still essential. For both ST and TEI, the threshold converges on a compulsory, output-anchored reflective checkpoint. Structure need not prescribe the entire practice process, but it must require a non-negotiable reflective product. In ST, this is enacted through targeted prompts that elicit higher-order strategic reasoning (e.g., S20, S21). In TEI, it is realized by binding technology-generated evidence (e.g., a performance video) to a required, analytic action, such as a time-stamped self-correction log or peer-annotated review (e.g., S5, S28). These elements are critical to maintain intervention–measurement alignment and safeguard the quality of reflection.

It is important to underscore that the MST articulated here is an empirically derived construct, not a precise, experimentally established cutoff. Its efficacy and optimal frequency of implementation require further validation. Future work should employ factorial designs that manipulate specific structuring components within ST and TEI (e.g., logbook present vs. absent; recording present vs. absent) to estimate boundary conditions, practical impacts, and sustainability of the MST across educational stages.

## 7. Future Research

Measurement–intervention fit is pivotal for accurately detecting effects. A recurrent issue is that interventions occur in musical tasks, yet assessments rely on generic cognitive tests or global teacher ratings, thereby underestimating true impact. Future studies should prioritize proximal, task-embedded measures—e.g., in-class observation, video annotation, or learner self-records—over sole reliance on distal EF tests, to capture authentic changes in SRL and metacognition. At the same time, for “high-structure but low effect” findings, it remains unclear whether the primary driver is measurement misfit or stage differences; studies within the same grade band that triangulate multiple measures in parallel are needed to disentangle these sources.

The heatmap generated in this study highlights substantial gaps in the distribution of intervention combinations across educational stages. Existing research has predominantly focused on SLS × ST or SLS × TEI at the university level, while systematic evidence for multi-mechanism integration (SLS + ST + TEI) is almost entirely lacking in primary and secondary school contexts. Future research should further compare the differential effects of single- versus multi-mechanism interventions across educational stages and examine whether these differences are attributable to learners’ cognitive developmental levels, the availability of teacher training resources, or institutional and curricular arrangements. It is also noteworthy that, within the present sample, no dyadic combination consisting solely of ST and TEI was observed. To address this gap, future studies could adopt experimental or quasi-experimental designs that manipulate SLS as an independent variable, establishing varying levels of structural conditions. Such designs would make it possible to test whether the combination of ST and TEI can still generate stable learning gains under conditions of no structure, partial structure, and high structure, thereby enabling a more rigorous evaluation of the necessity of SLS and the identification of the minimum structural threshold required to achieve effective intervention outcomes.

Lastly, non-formal education, professional musicians, and cross-stage samples are still under-studied. Although these areas are nearly blank on the heatmap, they could offer perspectives that formal schooling misses. For example, professionals’ SRL relies more on long-term habits and high-level strategic awareness—well suited to qualitative longitudinal or case designs to articulate “expert-type” SRL—whereas SRL in non-formal settings is more motivation- and autonomy-driven, inviting experimental or survey comparisons with formal contexts. Together, these directions can broaden and diversify our understanding of SRL and metacognition.

## Figures and Tables

**Table 1:** Reasons for Exclusion of Irrelevant Records by Database.

Database	Non-Empirical	Literature Review	Not Related to Music	Not Related to Educational Intervention	No Improvement in SRL/Metacognition
Scopus	40	25	100	107	88
WoS	25	22	67	95	145
ERIC	4	9	7	25	43
PsycINFO	8	9	22	29	80
Total	77	65	196	256	356

**Table 2:** Characteristics of Included Studies.

Study ID	Author (Year), Country	Method	Participants (*n*)	Educational Stage	SRL/Metacognition-Related Results	Intervention Type	Structuring Level	Risk of Bias
S1	Bentley et al. (2023), Australia	RCT (quantitative)	213	Preschool	Self-regulation improved across behavioral, cognitive, emotional, and classroom domains; gains maintained at follow-up.	SLS	High	Low
S2	Nielsen (2015), Norway	Multi-case study (qualitative)	2	University (undergraduate)	Advanced SRL processes observed, including planning, monitoring, strategy selection, and post-practice evaluation.	ST + TEI + SLS	Medium	Low
S3	Miksza et al. (2018), USA	Multiple-baseline intervention (mixed)	3	University (undergraduate)	Clear enhancements in goal setting, strategy variety, attentional control, and reflective monitoring, especially for initially lower-SRL learners.	TEI + SLS	High	Moderate
S4	Li et al. (2023), China	Experimental design (mixed)	84	University (undergraduate)	Metacognitive regulation improved; metacognition aligned with higher SRL readiness; positive behavioral changes noted.	TEI + ST + SLS	High	Low
S5	Boucher et al. (2020), Canada	Experiment (mixed)	16	University (undergraduate, CÉGEP level)	Video-based self-review promoted mature practice strategies and more deliberate self-monitoring.	TEI + SLS	Medium	Low
S6	Silveira and Gavin (2016), USA	Experiment (quantitative)	112	Secondary school	Recording and playback increased the accuracy and rigor of self-assessment, indicating stronger monitoring.	TEI + SLS	Medium	Low
S7	Zachariou et al. (2023), Cyprus	Quasi-experiment (quantitative)	117	Primary school	Teacher-rated self-regulation and metacognitive knowledge improved; classroom on-task metacognitive control showed no clear change.	ST + SLS	Medium	Low
S8	Song et al. (2024), The Netherlands	Within-subject experiment (quantitative)	50	Primary and secondary school	Robot-guided self-assessment boosted motivation and performance and reinforced self-monitoring.	TEI + SLS	Medium	Low
S9	Williams et al. (2023), Australia	RCT (quantitative)	213	Preschool	Self-regulation improved, most notably for emotional and behavioral regulation; executive-function outcomes were unchanged.	SLS	High	Moderate
S10	Wan et al. (2023), Australia	Multi-case study (qualitative)	4	Primary school (upper grades)	With sustained teacher scaffolding, digital tools fostered monitoring, self-evaluation, and strategy diversification; gains weakened without support.	TEI	Medium	Low
S11	Prichard (2021), USA	Quasi-experiment with control (mixed)	105	Secondary school	Strategy knowledge and observed strategy use increased; SRL ratings aligned closely with observed practice behavior.	ST + SLS	High	Low
S12	López-Íñiguez and McPherson (2020), Finland	Single-case intervention (mixed)	1	Professional musician	Growth in reflective practice, strategy complexity, and intrinsic motivation across performance cycles.	NA	NA	Moderate
S13	Liu and Liao (2025), China	Experimental (quantitative)	220	University (undergraduate)	Deeper learning motives strengthened and creative output improved; practice-strategy scores showed no short-term change.	TEI + SLS	High	Moderate
S14	Cheng et al. (2020), Hong Kong	Longitudinal (mixed)	74	University (undergraduate)	Self-monitoring, reflection, self-management, and learner autonomy increased on self-report and interviews.	ST + SLS	High	Moderate
S15	Bae et al. (2025), Korea	Quasi-experiment (quantitative)	96	University (undergraduate)	Cognitive strategies improved; metacognitive self-regulation showed no clear change.	SLS	Low	Low
S16	Allingham and Wöllner (2022), multi-country	Cross-sectional survey (quantitative)	256	University (undergraduate)	More frequent slow-practice use was associated with higher SRL.	NA	NA	Moderate
S17	Peistaraite and Clark (2020), multi-country	Cross-sectional survey (quantitative)	334	University students and professional musicians	Emotion reappraisal related positively to SRL; repression related negatively; contextual and gender differences noted.	NA	NA	Low
S18	Brook and Upitis (2015), Canada	Case study (mixed)	83	Private studios (children and adults)	Electronic portfolios supported SRL cycles when strongly integrated by teachers; benefits were limited with weak integration.	TEI + SLS	Medium	Low
S19	Hatfield (2016), Norway	Pre-post design (mixed)	6	University (undergraduate)	Gains in goal setting, self-observation, and self-evaluation were evident and sustained; worry decreased.	ST + SLS	High	Low
S20	Osborne et al. (2021), Australia	Multi-case study (qualitative)	7	University (postgraduate)	Structured OMMP prompts improved attentional focus, strategic planning, and post-performance evaluation.	ST + SLS	High	Moderate
S21	Woody (2023), USA	RCT (quantitative)	100	University (undergraduate)	Brief cognitive-skill prompts increased SRL-aligned behaviors and more positive practice appraisals.	ST	Medium	Low
S22	Capistrán-Gracia and Perakaki (2025), Mexico	Action research (qualitative)	18	University (undergraduate)	Time management, reflective thinking, and critical analysis improved under a flipped-classroom format.	ST + SLS	High	Moderate
S23	Mateos-Moreno et al. (2025), Spain	Action research (qualitative)	3	High school/pre-college	Reflective logs and guided questioning strengthened planning, monitoring, post-practice reflection, and attentional control; some fatigue reported.	ST	Medium	Moderate
S24	Prichard (2017), USA	Quasi-experiment (quantitative)	136	Secondary school	Strategy repertoire expanded and practice became more efficient; overall SRL composite remained largely unchanged.	ST + SLS	High	Moderate
S25	Zachariou and Bonneville-Roussy (2024), UK	Observational study (quantitative)	64	Primary school	Teacher autonomy support related positively to students’ SRL behaviors and negatively to SRL-failure behaviors.	NA	NA	High
S26	López-Calatayud and Tejada (2024), Spain	Multi-case study (qualitative)	4	Primary music academy	Real-time intonation feedback with structured logging increased self-monitoring, adaptive strategy use, and persistence.	TEI + SLS	High	Moderate
S27	Kegelaers and Oudejans (2020), The Netherlands	Mixed-method evaluation	15	University (undergraduate and professional musicians)	Awareness, goal-directed planning, and reflective habits increased; clear gains on ability measures were not demonstrated.	ST + SLS	High	Low
S28	Pike (2015), USA	Collective case (qualitative)	3	University (undergraduate, piano majors)	Online practicum normalized self-monitoring and reflective routines; self-evaluation and strategy adjustment improved.	TEI + SLS	Medium–High	Moderate
S29	Koner et al. (2024), USA	Pre-post experiment (quantitative)	117	High school	Self-efficacy improved; other SRL subscales showed mixed patterns.	ST + SLS	High	Moderate
S30	Feng (2024), China	Quasi-experiment (quantitative)	125	University (undergraduate)	Metacognitive functioning improved following an integrated voice and mental-health module.	ST + SLS	High	Moderate
S31	Utermohl de Queiroz et al. (2025), International	Collective case (qualitative)	12	Non-formal education	Teachers offered motivational and procedural cues; explicit elicitation of metacognitive reflection was limited.	TEI + ST + SLS	Medium	Moderate

**Table 3:** Educational Stage × Intervention Type.

Educational Stage	SLS	ST	TEI	SLS + ST	SLS + TEI	SLS + ST + TEI	Total
Preschool	2	0	0	0	0	0	2
Primary school	0	0	1	1	1	0	3
Secondary school	0	1	0	2	1	0	4
High school	0	0	0	1	0	0	1
Primary and Secondary	0	0	0	0	1	0	1
University	1	1	0	5	4	2	13
University and Professional musician	0	0	0	1	0	0	1
Non-formal education	0	0	0	0	1	1	2
Total	3	2	1	10	8	3	27

**Table 4:** Results of the Meta-Analysis.

	Author, (Year)	*N*	*d*	*g*	SE	95%CI_Low	95%CI_High	Weight	*z*_Value	*p*_Value
0	Bentley et al. (2023)	213	0.039354	0.039214	0.137234	−0.22976	0.308193	53.0978	0.285746	0.775072
1	Bentley et al. (2023), effect 2	213	0.031148	0.031037	0.137229	−0.23793	0.300006	53.1016	0.226169	0.82107
2	Bentley et al. (2023), effect 3	213	0.024544	0.024456	0.137226	−0.24451	0.293419	53.10402	0.178219	0.858551
3	Li et al. (2023)	84	0.55	0.544954	0.222475	0.108904	0.981004	20.20407	2.449511	0.014305
4	Li et al. (2023), effect 2	84	0.67	0.663853	0.224389	0.22405	1.103656	19.86078	2.958491	0.003091
5	Boucher et al. (2020)	16	0.79	0.746909	0.51714	−0.26668	1.760503	3.739247	1.444308	0.148652
6	Boucher et al. (2020), effect 2	16	0.85	0.803636	0.519791	−0.21515	1.822426	3.701206	1.546077	0.122086
7	Boucher et al. (2020), effect 3	16	0.72	0.680727	0.514277	−0.32726	1.68871	3.780991	1.323659	0.185616
8	Boucher et al. (2020), effect 4	16	0.66	0.624	0.512023	−0.37957	1.627566	3.814348	1.218694	0.22296
9	Boucher et al. (2020), effect 5	16	1.22	1.153455	0.539979	0.095097	2.211812	3.429628	2.136112	0.03267
10	Silveira and Gavin (2016)	112	0.67	0.665421	0.194142	0.284904	1.045939	26.53153	3.427503	0.000609
11	Silveira and Gavin (2016), effect 2	112	0.55	0.546241	0.192474	0.168992	0.923491	26.99322	2.837998	0.00454
12	Silveira and Gavin (2016), effect 3	112	0.81	0.804465	0.196478	0.419369	1.189561	25.90445	4.094437	4.23 × 10^−5^
13	Zachariou et al. (2023)	98	0.238209	0.236343	0.203409	−0.16234	0.635024	24.1691	1.161912	0.245271
14	Zachariou et al. (2023), effect 2	98	0.088469	0.087776	0.202804	−0.30972	0.485271	24.31348	0.43281	0.665153
15	Zachariou et al. (2023), effect 3	98	0.098176	0.097407	0.202826	−0.30013	0.494946	24.3081	0.480246	0.631053
16	Prichard (2021)	105	2.27	2.253431	0.249558	1.764296	2.742565	16.05667	9.029672	0
17	Prichard (2021), effect 2	105	1.72	1.707445	0.227994	1.260576	2.154314	19.23765	7.488982	6.95 × 10^−14^
18	Bae et al. (2025)	96	0.51	0.50592	0.207539	0.099145	0.912695	23.21682	2.437716	0.01478
19	Bae et al. (2025), effect 2	96	0.49	0.48608	0.207291	0.079789	0.892371	23.2722	2.344912	0.019032
20	Bae et al. (2025), effect 3	96	0.42	0.41664	0.206502	0.011895	0.821385	23.45038	2.017604	0.043633
21	Bae et al. (2025), effect 4	96	0.13	0.12896	0.204513	−0.27189	0.529806	23.90872	0.63057	0.528322

## Data Availability

No new data were created or analyzed in this study. Data sharing is not applicable to this article.

## Supplementary Materials

The following supporting information can be downloaded at: https://www.mdpi.com/article/10.3390/jintelligence13120162/s1, Table S1: The search strings; Table S2: Full-text Exclusion List. Table S3: Measurement Instruments and Fit with SRL Interventions.
