# Supplementary material for: From Practice to Reflection: A Systematic Review of Mechanisms Driving Metacognition and SRL in Music
Source: J Intell. 2025 Dec 9;13(12):162. doi: 10.3390/jintelligence13120162 (PMC12734040; doi:10.3390/jintelligence13120162)
Supplement: Supplementary file 1 [file jintelligence-13-00162-s001.zip › jintelligence-3876251-supplementary.pdf]

Table S1. The search strings.

Scopus	TITLE-ABS-KEY (education OR instruction OR teaching OR learning OR classroom OR training OR strategy OR pedagogy OR curriculum) AND ("learning regulation" OR "self-regulated learning" OR "self-regulation" OR "SRL" OR "goal setting" OR "cognitive regulation" OR "time management" OR "learning strateg*" OR "motivational regulation" OR metacogniti* OR "metacognitive strategy" OR "self-monitoring")
ERIC	https://eric.ed.gov/?q=music+AND+%28education+OR+instruction+OR+teaching+OR+learning+OR+classroom+OR+training+OR+strategy+OR+pedagogy+OR+curriculum%29+AND+%28%22learning+regulation%22+OR+%22self-regulated+learning%22+OR+%22self-regulation%22+OR+%22SRL%22+OR+%22goal+setting%22+OR+%22cognitive+regulation%22+OR+%22time+management%22+OR+%22learning+strateg*%22+OR+%22motivational+regulation%22+OR+metacogniti*+OR+%22metacognitive+strategy%22+OR+%22self-monitoring%22%29
Web of Science	music AND (education OR instruction OR teaching OR learning OR classroom OR training OR strategy OR pedagogy OR curriculum) AND ("learning regulation" OR "self-regulated learning" OR "self-regulation" OR "SRL" OR "goal setting" OR "cognitive regulation" OR "time management" OR "learning strateg*" OR "motivational regulation" OR metacogniti* OR "metacognitive strategy" OR "self-monitoring") (topic)
PsycINFO	TI (music AND (education OR instruction OR teaching OR learning OR classroom OR training OR strategy OR pedagogy OR curriculum) AND ("learning regulation" OR "self-regulated learning" OR "self-regulation" OR "SRL" OR "goal setting" OR "cognitive regulation" OR "time management" OR "learning strateg*" OR "motivational regulation" OR metacogniti* OR "metacognitive strategy" OR "self-monitoring")) OR SU (music AND (education OR instruction OR teaching OR learning OR classroom OR training OR strategy OR pedagogy OR curriculum) AND ("learning regulation" OR "self-regulated learning" OR "self-regulation" OR "SRL" OR "goal setting" OR "cognitive regulation" OR "time management" OR "learning strateg*" OR "motivational regulation" OR metacogniti* OR "metacognitive strategy" OR "self-monitoring")) OR AB (music AND (education OR instruction OR teaching OR learning OR classroom OR training OR strategy OR pedagogy OR curriculum) AND ("learning regulation" OR "self-regulated learning" OR "self-regulation" OR "SRL" OR "goal setting" OR "cognitive regulation" OR "time management" OR "learning strateg*" OR "motivational regulation" OR metacogniti* OR "metacognitive strategy" OR "self-monitoring"))

Table S2. Full-text Exclusion List (n = 65)

Number	Title
**1**	A translational application of music for preschool cognitive development:Rct evidence for improved executive function, self-regulation, and school readiness
**2**	Learning Pre-Played Solos: Self-Regulated Learning Strategies in Jazz/Improvised Music
**3**	Self-regulated music practice: Microanalysis as a data collection technique and inspiration for pedagogical intervention.
**4**	The effects of musical feedback training on metacognition and self-directed learning.
**5**	Video feedback and the choice of strategies of college-level guitarists during individual practice.
**6**	The effect of audio recording and playback on self-assessment among middle school instrumental music students.
**7**	Exploring the effects of a musical play intervention on young children’s self-regulation and metacognition.
**8**	‘How would you score yourself?’: The effect of self-assessment strategy through robots on children’s motivation and performance in piano practice.
**9**	Rhythm and movement delivered by teachers supports self-regulation skills of preschool-aged children in disadvantaged communities: A clustered RCT.
**10**	Digital listening tools to facilitate children’s self-regulation of instrumental music practice.
**11**	The impact of music practice instruction on middle school band students’ independent practice behaviors.
**12**	Applying self-regulated learning and self-determination theory to optimize theperformance of a concert cellist
**13**	Integrating IBM Watson BEAT Generative AI Software into Flute Music Learning: The Impact of Advanced AI Tools on Students' Learning Strategies

**14**	Learner Autonomy in Music Performance Practices
**15**	The effect of a practice checklist on performance achievement, practice time, focal practice areas, and practice strategies of collegiate non-music majors enrolled in a beginning "Danso," Korean traditional wind instrument class
**16**	Slow practice and tempo-management strategies in instrumental music learning: Investigating prevalence and cognitive functions
**17**	Emotion Regulation Processes Can Benefit Self-Regulated Learning in Classical Musicians
**18**	Can an online tool support contemporary independent music teaching and learning?
**19**	Digital Listening Tools to Facilitate Children's Self-Regulation of Instrumental Music Practice
**20**	Performing at the Top of One's Musical Game
**21**	Self-Regulated Music Practice: Microanalysis as a Data Collection Technique and Inspiration for Pedagogical Intervention
**22**	Exploring the effects of a musical play intervention on young children's self-regulation and metacognition
**23**	Using a microanalysis intervention to examine shifts in musicians’ self-regulatedlearning.
**24**	Using a microanalysis intervention to examine shifts in musicians' self-regulated learning
**25**	Using a microanalysis intervention to examine shifts in musicians’ self-regulated learning
**26**	University musicians' use of component cognitive skills in practice: A self-report study
**27**	Exploring self-regulation through a reflective practicum: A case study of improvement through mindful piano practice
**28**	A Seminar on Music Research in Higher Education and the Flipped Classroom: An Action Research Project
**29**	Teaching to Practice Productively and Consciously: An Action-Research Study in One-to-One Instrumental Music Teaching

**30**	Music Practice Instruction in Middle School Instrumental Ensembles: An Exploratory Analysis
**31**	The Impact of Music Practice Instruction on Middle School Band Students' Independent Practice Behaviors
**32**	The role of autonomy support from teachers in young learners' self-regulation in dyadic contexts: An examination through three-level multilevel analysis
**33**	The effects of musical feedback training on metacognition and self-directed learning
**34**	'How Would you Score Yourself?': The Effect of Self-assessment Strategy Through Robots on Children's Motivation and Performance in Piano Practice
**35**	A translational application of music for preschool cognitive development: RCT evidence for improved executive function, self-regulation, and school readiness
**36**	Video feedback and the choice of strategies of college-level guitarists during individual practice
**37**	Rhythm and movement delivered by teachers supports self-regulation skills of preschool-aged children in disadvantaged communities: A clustered RCT
**38**	The Effect of Audio Recording and Playback on Self-Assessment Among Middle School Instrumental Music Students
**39**	Bentley, L. A., et al. (2023). A Translational Application of Music for Preschool Cognitive Development: RCT Evidence for Improved Executive Function, Self-Regulation, and School Readiness
**40**	Self-Regulation Strategies and Behaviors in the Initial Learning of the Viola and Violin with the Support of Software for Real-Time Instrumental Intonation Assessment
**41**	Exploring Self-Regulation through a Reflective Practicum: A Case Study of Improvement through Mindful Piano Practice.
**42**	The Impact of Music Practice Instruction on Middle-School Band Students' Independent Practice Behaviors.

**43**	Exploring the Effects of a Musical-Play Intervention on Young Children's Self-Regulation and Metacognition.
**44**	A Seminar on Music Research in Higher Education and the Flipped Classroom: An Action Research Project
**45**	Exploring the effects of a musical play intervention on young children's self-regulation and metacognition
**46**	The role of autonomy support from teachers in young learners' self-regulation in dyadic contexts: An examination through three-level multilevel analysis
**47**	A translational application of music for preschool cognitive development: RCT evidence for improved executive function, self-regulation, and school readiness
**48**	The effects of musical feedback training on metacognition and self-directed learning
**49**	Rhythm and movement delivered by teachers supports self-regulation skills of preschool-aged children in disadvantaged communities: A clustered RCT
**50**	Using a microanalysis intervention to examine shifts in musicians' self-regulated learning
**51**	Digital Listening Tools to Facilitate Children's Self-Regulation of Instrumental Music Practice
**52**	University musicians' use of component cognitive skills in practice: A self-report study
**53**	Self-Regulated Music Practice: Microanalysis as a Data Collection Technique and Inspiration for Pedagogical Intervention
**54**	A Process Evaluation of a Performance Psychology Intervention for Transitioning Elite and Elite Musicians
**55**	The Impact of Music Practice Instruction on Middle School Band Students' Independent Practice Behaviors
**56**	Video feedback and the choice of strategies of college-level guitarists during individual practice
**57**	Performing at the top of one's musical game
**58**	Can an online tool support contemporary independent music teaching and learning?

**59**	Exploring self-regulation through a reflective practicum: a case study of improvement through mindful piano practice
**60**	Using a synchronous online teaching internship to develop pedagogical skills and explore teacher identity: A case study
**61**	The Impact of Mindfulness Techniques on Self-Regulation of Practice Habits and Routines of High School Instrumental Students.
**62**	Exploring the Integration of College Vocal Music Program and College Students' Mental Health Education in the Context of Big Data
**63**	Investigating if and how string teachers instruct and support the self-regulation of students' practice in online lessons
**64**	Investigating if and how string teachers instruct and support the self-regulation of students' practice in online lessons
**65**	Can an online tool support contemporary independent music teaching and learning?

Table S3. Measurement Instruments and Fit with SRL Interventions

No.	Study Title	Measurement Instruments	SRL / Metacognition/ Both	Measurement–Intervention Fit
**S1**	A transtational application of music for preschool cognitive development:Rct evidence for improved executive function, self-regulation, and school readiness	CSBQ, EF composite (Go/No-Go, Mr Ant, Card Sort), Teacher interviews	SRL	Intermediate
**S2**	Learning Pre-Played Solos: Self-Regulated Learning Strategies in Jazz/Improvised Music	Stimulated recall interviews + video-recorded individual practice + classroom observation	Both	Proximal

**S3**	Using a Microanalysis Intervention to Examine Shifts in Musicians' Self-Regulated Learning	Structured SRL Microanalysis Protocol (pre/mid/post practice reflection)	Both	Proximal
**S4**	The Effects of Musical Feedback Training on Metacognition and Self-Directed Learning	Musical Learning Metacognition Questionnaire; Self-Directed Learning Readiness Scale	Both	Intermediate
**S5**	Video Feedback and the Choice of Strategies of College-Level Guitarists During Individual Practice	Practice logs + strategy use coding + video playback reflection	SRL	Proximal
**S6**	The Effect of Audio Recording and Playback on Self-Assessment Among Middle School Instrumental Music Students	Self-evaluation rubrics + teacher ratings (with audio playback)	Metacognition	Proximal
**S7**	Exploring the Effects of a Musical Play Intervention on Young Children's Self-Regulation and Metacognition	a) In-task observation (Train Track Task); b) Metacognitive interview; c) CHILD Teacher Rating Scale	Both	Intermediate
**S8**	"How Would You Score Yourself?": The Effect of Self-assessment Strategy Through Robots on Children's Motivation and Performance in Piano Practice	Robot-facilitated self-scoring (pitch, rhythm, tempo) + motivation questionnaires + teacher ratings	Both	Intermediate
**S9**	'How Would You Score Yourself?': The Effect of Self-Assessment Strategy Through Robots on Children's Motivation	Robot-facilitated self-rating (pitch, rhythm, tempo); Motivation questionnaire	Both	Distal

	and Performance in Piano Practice			
**S10**	Digital listening tools and children's self-regulation in instrumental music practice	Video-coded practice observations; semi-structured student interviews (×2); teacher interviews (×3); member checking	Both	Proximal
**S11**	The Impact of Music Practice Instruction on Middle School Band Students' Independent Practice Behaviors	Strategy listing (free recall); video observation of practice; SRL rating scale (Likert-type)	SRL	Proximal
**S12**	Applying self-regulated learning and self-determination theory to optimize the performance of a concert cellist	Likert scales; dichotomous items; reflective diaries (content analysis, Leximancer); regression analyses	Both	Intermediate
**S13**	Integrating IBM Watson BEAT generative AI software into flute music learning: the impact of advanced AI tools on students' learning strategies	R-SPQ-2F (Deep/Surface Motive & Strategy); teacher ratings of composition/interpretation	SRL	Distal
**S14**	Learner Autonomy in Music Performance Practices	Self-report questionnaire (17 transferable skills); semi-structured interviews	Both	Intermediate
**S15**	The effect of a practice checklist on performance achievement, practice time, focal practice areas, and practice strategies of collegiate non-music majors enrolled in a beginning "Danso" class	Adapted MSLQ (cognitive, metacognitive, time management); self-reported practice time/foci; performance ratings	Both	Intermediate

**S16**	Slow practice and tempo-management strategies in instrumental music learning: Investigating prevalence and cognitive functions	MSRL questionnaire; self-reported slow-practice frequency and purposes	SRL	Intermediate
**S17**	Emotion regulation processes can benefit self-regulated learning in classical musicians	SRLMQ (12-dimension SRL); ERQ (Emotion Regulation Questionnaire)	SRL	Distal
**S18**	Can an online tool support contemporary independent music teaching and learning?	Semi-structured teacher interviews; CSLP-based student questionnaire; classroom observations; e-portfolio/platform usage logs	Both	Intermediate
**S19**	Performing at the top of one's musical game	SLMQ (Zimmerman model, 13 subscales); semi-structured interviews; study/practice logs; iPad MusicJournal records	Both	Proximal
**S20**	Using a microanalysis intervention to examine shifts in musicians' self-regulated learning	OMMP reflective diary; video-stimulated recall interviews; performance video review; thematic analysis notes	Both	Proximal
**S21**	University musicians' use of component cognitive skills in practice: A self-report study	Researcher-designed practice response sheet with open-ended prompts, quantified via a 7-category coding guide based on content analysis.;Moderate	SRL	Intermediate
**S22**	A Seminar on Music Research in Higher Education and the Flipped Classroom: An Action Research Project	Ad hoc questionnaire, reflective diary, field notes.	Both	Proximal

**S23**	Teaching to practice productively and consciously: an action-research study in one-to-one instrumental music teaching	Teacher logs; student practice logs (per-session); pre/post semi-structured interviews (MaxQDA coding)	Both	Proximal
**S24**	Music Practice Instruction in Middle School Instrumental Ensembles: An Exploratory Analysis	Practice strategy checklist; 20-min independent-practice video coding (frames, strategy frequency); observational SRL rating	SRL	Proximal
**S25**	The role of autonomy support from teachers in young learners' self-regulation in dyadic contexts: An examination through three-level multilevel analysis	Classroom video coding: Whipple teacher autonomy-support scale; Whitebread positive SRL codes; Bryce & Whitebread SRL-failure codes	Both	Intermediate
**S26**	Self-regulation strategies and behaviors in the initial learning of the viola and violin with real-time intonation assessment software	Plectrus software feedback/score logs; adapted Austin & Berg (2006) practice diary; practice video analysis; parent interviews	Both	Proximal
**S27**	A Process Evaluation of a Performance Psychology Intervention for Transitioning Elite and Elite Musicians	Semi-structured interviews; monitoring logs; open-ended responses; Likert workshop and overall intervention evaluations	Both	Intermediate
**S28**	Using a Synchronous Online Teaching Internship to Develop Pedagogical Skills and Explore Teacher Identity	Classroom video recordings with annotations; written reflections; focus-group interviews (constant comparative analysis)	Both	Intermediate

**S29**	The Impact of Mindfulness Techniques on Self-Regulation of Practice Habits and Routines of High School Instrumental Students	Miksza (2012) SRL questionnaire (Self-efficacy, Method, Behavior, Time management, Social influences); weekly reflection sheets	SRL	Intermediate
**S30**	Exploring the Integration of College Vocal Music Program and College Students' Mental Health Education in the Context of Big Data	Metacognitive Functioning Scale; SCL-90 (and total score)	Metacognition	Distal
**S31**	Investigating if and how string teachers instruct and support the self-regulation of students' practice in online lessons	Lesson observations (video + field notes); semi-structured interviews; teaching-materials analysis (descriptive statistics)	Both	Intermediate
